# Parental awareness and utilization of meningococcal serogroup B vaccines in the United States

**DOI:** 10.1186/s12889-020-09181-8

**Published:** 2020-07-14

**Authors:** Amit Srivastava, Amanda Dempsey, Alex Galitsky, Mansour Fahimi, Liping Huang

**Affiliations:** 1 Vaccine Medical Development, Scientific & Clinical Affairs, Pfizer Inc, 300 Technology Square, 3rd Floor, Cambridge, MA 02139 USA; 2 University of Colorado, Denver, CO USA; 3 Ipsos, New York, NY USA; 4 Vaccine Medical Development, Scientific & Clinical Affairs, Pfizer Inc,, Collegeville, PA USA

**Keywords:** Meningococcal disease, Vaccination, Meningococcal serogroup B, Category B recommendation, Socioeconomic disparities, Racial disparities, Vaccine awareness, Adolescents

## Abstract

**Background:**

Meningococcal serogroup B (MenB) is the most common cause of invasive meningococcal disease (IMD) in the United States. The US Advisory Committee on Immunization Practices (ACIP) recommends vaccination of healthy adolescents against MenB based on shared clinical decision-making (Category B recommendation). This survey assessed factors associated with MenB vaccine awareness, utilization, and interest among parents/guardians of US adolescents.

**Methods:**

Survey participants were identified in 2016 through KnowledgePanel®, an online random sample of US households; population-based weighting methodology was used to ensure data reflected a demographically representative population sample. Adults with ≥1 dependent aged 16–19 years were eligible and completed an online questionnaire. Respondents were grouped in terms of MenB vaccination of their child as: 1) vaccinated, 2) intending to vaccinate, 3) MenB vaccine-unaware, or 4) vaccine-aware but not intending to vaccinate. Univariate and multivariate analyses were used to identify factors influencing MenB vaccine awareness and utilization; univariate analyses used the weighted proportion of each group or weighted means, and multivariate analyses used logistic regression models based on the weighted study sample of each group.

**Results:**

Six hundred nineteen parents/guardians participated, corresponding to 26,266,700 members of the US population after weighting. MenB vaccine awareness was significantly associated with parent race and sex. Specifically, 57% of parents were unaware of MenB vaccines, and there was significantly higher lack of awareness among males and those of Hispanic or non-White ethnicity. In addition, 36% of unaware parents/guardians were interested in and seeking MenB vaccine information from their healthcare provider (HCP), and there was higher interest among parents of Hispanic ethnicity. ‘Vaccinated/intending to vaccinate’ versus ‘not intending to vaccinate’ and ‘vaccinated’ versus ‘intending to vaccinate’ were both strongly associated with whether an HCP had recommended vaccination (odds ratios, 4.81 [95% CI 2.46, 9.35] and 5.66 [95% CI 2.46, 12.87], respectively).

**Conclusions:**

Racial and socioeconomic disparities exist in the awareness and utilization of MenB vaccines among parents/guardians of US adolescents. HCP discussion and recommendation are critical catalysts for MenB vaccination and underscore the need to accurately interpret and implement the shared clinical decision-making (Category B) recommendation.

## Background

Invasive meningococcal disease (IMD) is an uncommon and unpredictable infection that progresses rapidly and can have severe consequences, including death and disfigurement, despite appropriate medical treatment [1–5]. In the United States, serogroup B (MenB) is now the leading cause of IMD among the 6 common meningococcal disease-causing serogroups globally (ie, A, B, C, W, X, and Y) [6, 7]. In addition to infants, adolescents and young adults are particularly vulnerable because they are the primary asymptomatic carriers, with meningococcal carriage peaking at around 19 years of age [8]. In institutional settings such as college dormitories, carriage rates can exceed 50% [9, 10] and can lead to disease transmission due to age-typical social mixing behaviors [7]. The Centers for Disease Control and Prevention (CDC) Enhanced Meningococcal Surveillance has shown that MenB accounts for approximately 69.6% of IMD cases in 16- to 23-year-olds in the United States and MenB has caused all 14 meningococcal outbreaks on US college campuses since 2011 [6, 11–13]. MenB vaccines have been available in the United States since 2014, which currently include MenB-FHbp (Trumenba®, Pfizer Inc, Philadelphia, PA) and MenB-4C (Bexsero®, GlaxoSmithKline Vaccines, Srl, Sovicille, Italy) [14, 15]. Quadrivalent conjugate MenACWY vaccines have been available in the United States since 2005 [16].

The US Advisory Committee on Immunization Practices (ACIP) recommends that all individuals receive MenACWY vaccine at 11 to 12 years of age, followed by a booster dose at 16 years of age [16, 17], which is a “Category A” or routine recommendation that applies to everyone in the indicated age group [18]. For MenB vaccines, ACIP issued a “Category B” recommendation (shared clinical decision-making in consultation with a healthcare provider [HCP] [19]) for vaccination of healthy individuals 16 to 23 years of age, with a preferred age of 16 to 18 years [20, 21]; a Category A recommendation was made for individuals ≥10 years of age at increased risk of IMD, such as those with asplenia or complement deficiency, or due to laboratory or outbreak exposure [20]. MenACWY vaccines were first recommended in 2005 followed by a booster dose recommendation in 2010 [16]. In 2018, MenACWY vaccination rates among individuals 13 to 17 years of age were 86.6% for ≥1 dose and 50.8% for ≥2 doses [22]. Since the initial recommendations for MenB vaccines in 2015 [21], the MenB vaccine uptake reported in CDC’s 2018 National Immunization Survey–Teen (NIS-Teen) indicated that 17.2% of US 17-year-olds had received ≥1 dose of a MenB multi-dose vaccine series (and < 50% complete the full series) [20, 22, 23].

Given that there is a structural difference in ACIP recommendations for the 2 classes of vaccines (MenACWY and MenB) needed for prevention of IMD among healthy adolescents [14, 15, 20], and MenB vaccine uptake in adolescents is low nationally [22], this study examined factors associated with parental/guardian awareness and utilization of MenB vaccines.

## Methods

### Recruitment and data collection

All data were collected via a survey conducted across the United States from December 9, 2016, through December 28, 2016. Survey participants were identified through the Ipsos KnowledgePanel® (formerly Growth from Knowledge; New York, NY), an online, probability-based, representative, random sample of US households. Because KnowledgePanel® includes households regardless of internet access, members are provided with laptops and/or internet access as needed for potential survey participation. This panel has been used for several previous national studies on immunization issues [24–27].

Email invitations were sent to a random, nationally representative sample of KnowledgePanel® households without details of the research topic, so as to minimize bias in the responding sample. Eligible participants were adult parents or guardians of ≥1 dependent aged 16 to 19 years, spoke either English or Spanish, and agreed with the confidentiality statement. An English or Spanish version of the survey was available to participants. Recruitment was quota oriented rather than a convenience sample. To have sufficient numbers and a proper distribution of parents whose adolescents were vaccinated or not vaccinated, and based on the MenB vaccination rate in 2015, the study planned to recruit at least 525 participants. These included 75 parents/guardians of MenB-vaccinated adolescents and 150 participants in each of the 3 additional mutually exclusive groups of varying levels of MenB awareness and utilization (i.e., MenB vaccine-unaware, aware and intending to vaccinate, or aware but not intending to vaccinate).

After completing screening questions, qualified participants completed a 25-min, self-administered online questionnaire, which included questions on demographics, vaccine awareness, status and intention, perceptions related to diseases and vaccines, HCP interaction related to vaccines, their decision-making process for vaccination, and general knowledge of the subject area (questionnaire available upon request). Respondents who indicated that their dependent(s) had been vaccinated with ≥1 dose of a MenB vaccine were then directed to a verification section requesting confirmation of the vaccine used and the date received (acquired via electronic medical records [EMRs], if available, or by contacting the HCP office). Additional demographic (age, race), social (education, housing), and economic data (insurance coverage) were extracted from KnowledgePanel® member profiles. All KnowledgePanel® members who participated in this study provided explicit consent prior to collection of any health/sensitive information.

### Groups and assessments

In the survey, respondents were first asked if they were aware of any available vaccines for meningococcal disease (meningitis). If they responded “yes,” they were prompted to indicate their awareness level for each vaccine type (i.e., MenB, MenACWY, and MenCY, the latter of which is recently no longer available in the United States) as “not at all aware,” “slightly aware,” “somewhat aware,” “moderately aware,” or “extremely aware.” “Aware” was defined as respondents who were at least slightly aware of MenB vaccines; otherwise, they were classified as “unaware.” Of those classified as “aware,” respondents were further designated as “vaccinated” if at least 1 of their children between 16 and 19 years of age had received ≥1 dose of a MenB vaccine, and as “intend to vaccinate” or “not intend to vaccinate” if they did or did not anticipate vaccinating their eligible children with MenB in the next 6 to 12 months, respectively. Respondents classified as “unaware” were asked to indicate their interest level for obtaining more information or speaking with an HCP about MenB vaccination, and vaccinating their child against MenB upon physician recommendation; the scale of interest level ranged from 1 to 7, where 1 indicated “not at all interested” and 7 indicated “extremely interested.” “Unaware but interested in vaccination” included respondents who were not aware of any MenB vaccine but responded that they were very interested (rated “6” or “7”).

### Data analysis

Data were analyzed using a population-based weighting method by adjusting sample weights to known population distributions among individuals ≥35 years of age and based on information retrieved from the US Census Bureau’s Current Population Survey (CPS; March 2016 Supplement). Specifically, computation was conducted on the design or base weights to reflect selection probabilities. Sample weights for all respondents (eligible and not eligible) were then adjusted to known population distributions obtained from the CPS. Prespecified dimensions used for weighting were sex, race/ethnicity, geographic region, educational attainment, household income, and language proficiency, consistent with prior studies [25–27].

Univariate and multivariate analyses were conducted to identify factors associated with MenB awareness, utilization, and interest. Specifically, 4 sets of comparisons were conducted: 1) aware versus unaware of the MenB vaccines, 2) aware and vaccinated or with intention to vaccinate versus aware with no intention to vaccinate, 3) aware and vaccinated versus aware with intention to vaccinate, and 4) unaware but interested in vaccination versus unaware and not interested in vaccination. Variables examined included age, sex, race (White, non-Hispanic; Hispanic; Black and Others, Non-Hispanic), education (high school or below, some college or above), property ownership (own, rent), annual income, insurance status (employer-based, Medicaid, no insurance, others), awareness of MenACWY vaccine, awareness of MenB outbreaks, seeing the same HCP, feeling the HCP knows their child well, HCP recommended MenB vaccine, and first made aware of MenB vaccine by HCP. For the univariate analyses, binary variables (e.g., yes, no) were presented by weighted proportion of each group. Continuous variables (e.g., number of children aged 16–19 years, household income) were presented by weighted means. For the multivariate analyses, logistic regression models based on weighted study samples were applied for each of the comparison groups. To ensure that results were consistent and robust, a classification and regression tree (CART) analysis was then used to identify key predictors and their estimated relative importance. CART is a robust procedure, particularly in the presence of multi-collinearity among a long list of predictor variables [28].

The analyses of how respondents became aware of MenB vaccines, if they received recommendation from their HCP, and the type of HCP who recommended MenB vaccines, were further compared across the 4 comparison groups. *P* values of the comparisons between 2 groups were calculated using a Chi-squared test of the weighted sample.

Univariate and multivariate analyses were conducted using the Statistical Analysis System statistical software package (version 9.4) and CART analysis was performed using Salford Predictive Modeler 8.2.

## Results

### Participants and weighted population sample

**Fig. 1:**
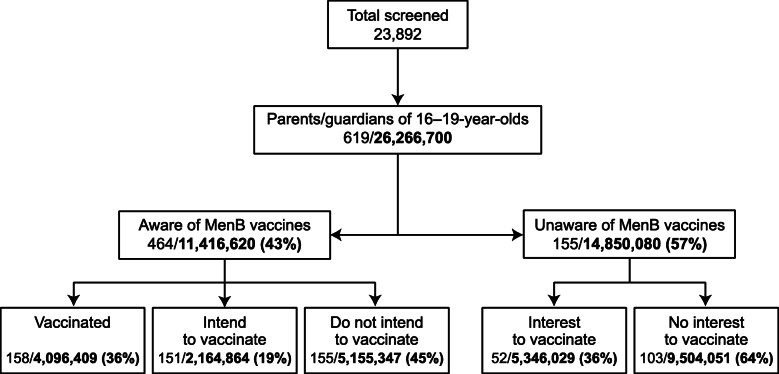
Flow chart of study population and groups. Shown are the number of respondents and the corresponding weighted number of the US population (weighted % based on US population distribution). MenB = meningococcal serogroup B. Among the respondents who indicated that they had vaccinated their adolescents, 57 of vaccinations were verified by either electronic medical records or by the healthcare provider

A total of 23,892 adults were screened and 619 were eligible and participated in the study (Fig. 1). Consistent with the pre-specified quotas for each parent category, of those eligible, 158 were parents/guardians of MenB-vaccinated adolescents, 151 were parents/guardians of unvaccinated adolescents who intended to vaccinate, 155 were parents/guardians of unvaccinated adolescents who were unaware of the MenB vaccines, and 155 were parents/guardians of unvaccinated adolescents who were aware of the MenB vaccines but did not intend to vaccinate (Table 1). Of note, as this was a quota sample, it was not possible to calculate a response rate. Among the 158 respondents who indicated that they had vaccinated their adolescents, 57 vaccinations were verified by either EMRs or the HCP. Of the 155 respondents who were unaware of MenB vaccines, 34% expressed interest in vaccination. Respondents were parents or guardians to ≥1 adolescent, and a majority had at least some college education, were 35 to 54 years of age, female, White or non-Hispanic, owned their homes, and/or had employer-based insurance.

**Table 1:** Weighted results of demographics, socioeconomic status, and access to care across MenB awareness and utilization

	Aware of MenB Vaccines (*n* = 467)	Not Aware of MenB Vaccines (*n* = 155)	Aware of and Vaccinated/Intend to Vaccinate (*n* = 312)	Aware of but Do Not Intend to Vaccinate (*n* = 155)	Aware of and Vaccinated (*n* = 161)	Aware of and Intend to Vaccinate (*n* = 151)	Unaware of but Interested (*n* = 52)	Unaware of but Not Interested (*n* = 103)
Weighted percentage^a^	43%	57%	55%	45%	66%	34%	36%	64%
Female parents/ guardians	62%	44%	62%	61%	66%	55%	46%	36%
Parents’ age group								
35–44 y	38%	40%	37%	41%	36%	37%	36%	55%
45–54 y	47%	44%	48%	45%	48%	46%	46%	35%
55–64 y	13%	15%	14%	11%	13%	15%	16%	9%
≥ 65 y	2%	1%	2%	3%	2%	1%	2%	0%
Number of children aged 16–19 y	1.24	1.26	1.25	1.23	1.27	1.20	1.30	1.10
Education: some college or above	61%	47%	63%	57%	64%	61%	46%	46%
Race/ethnicity								
White, non-Hispanic	60%	42%	56%	65%	56%	55%	39%	56%
Black, non-Hispanic	12%	14%	14%	10%	15%	10%	12%	19%
Hispanic	23%	34%	22%	23%	20%	26%	39%	15%
Other	5%	10%	8%	2%	8%	8%	11%	11%
Housing								
Own	74%	61%	76%	72%	78%	72%	58%	73%
Rent	23%	36%	23%	23%	21%	27%	38%	26%
Rent but no charge	3%	3%	1%	5%	1%	1%	3%	1%
Average annual income	$81 k	$72 k	$84 k	$76 k	$85 k	$83 k	$71 k	$75 k
Insurance								
Employer-based	63%	56%	66%	60%	68%	63%	55%	57%
Medicaid	12%	19%	7%	17%	6%	8%	21%	8%
Other	13%	17%	15%	11%	16%	12%	16%	23%
None	12%	9%	12%	11%	10%	16%	8%	12%
HCP factors								
Think HCP knows you or your child well (yes)	83%	73%	89%	76%	88%	88%	75%	64%
Generally see same HCP (yes)	89%	83%	89%	89%	88%	92%	84%	77%
Awareness								
Aware of MenACWY vaccine (yes)	29%	9%	34%	24%	38%	25%	10%	5%
Aware of MenB outbreaks (yes)	16%	8%	19%	12%	19%	19%	10%	0%

*HCP* healthcare provider, *MenACWY* meningococcal serogroups A, C, W, and Y, *MenB* meningococcal serogroup B

^a^All percentages included in the table refer to weighted percentage

The numbers and percentages of the US population estimated to correspond with numbers of participants in each group were also determined (Fig. 1). Of the 26,266,700 individuals in the full US population represented by the 619 respondents, an estimated 43% were aware of MenB vaccines; of these individuals, 36% had their adolescents vaccinated, 19% intended to vaccinate, and 45% did not intend to vaccinate. Of the estimated 57% who were not aware of the vaccine, an estimated 36% expressed interest in vaccination.

### Findings by comparison group

#### Aware versus not aware of MenB vaccines

Results from univariate analyses indicated that awareness of MenB vaccines was generally higher among parents who were female, White/non-Hispanic, covered by employer-based medical insurance, and aware of MenACWY vaccines or MenB outbreaks, as well as those who had higher educational attainment (college or above), owned their own home, or felt that their HCPs knew their child well, compared with parents who were unaware of the MenB vaccine (Table 1).

**Fig. 2:**
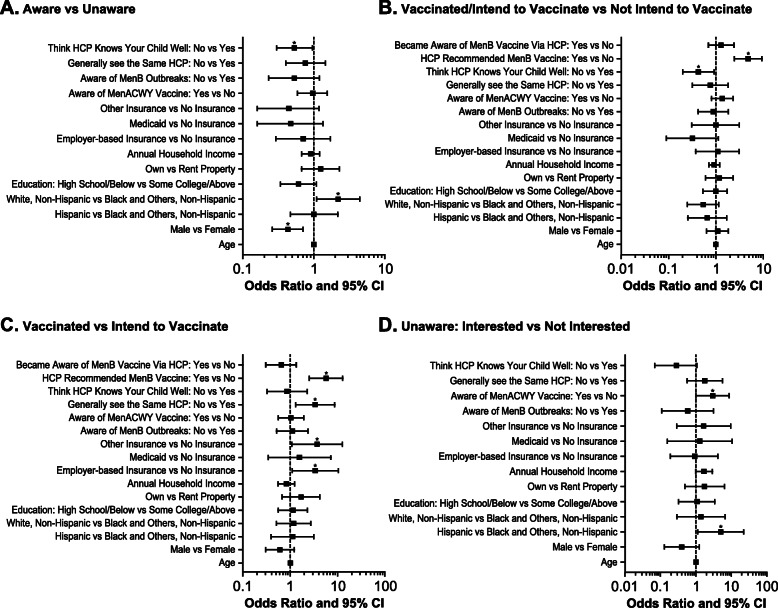
Factors associated with vaccine awareness and utilization according to logistic regression modeling. Shown are multivariate odds ratios with associated 95% CIs for MenB vaccine awareness and utilization. HCP = healthcare provider; MenACWY = meningococcal serogroups A, C, W, and Y; MenB = meningococcal serogroup B. *Denotes statistical significance with *P* < 0.05

**Fig. 3:**
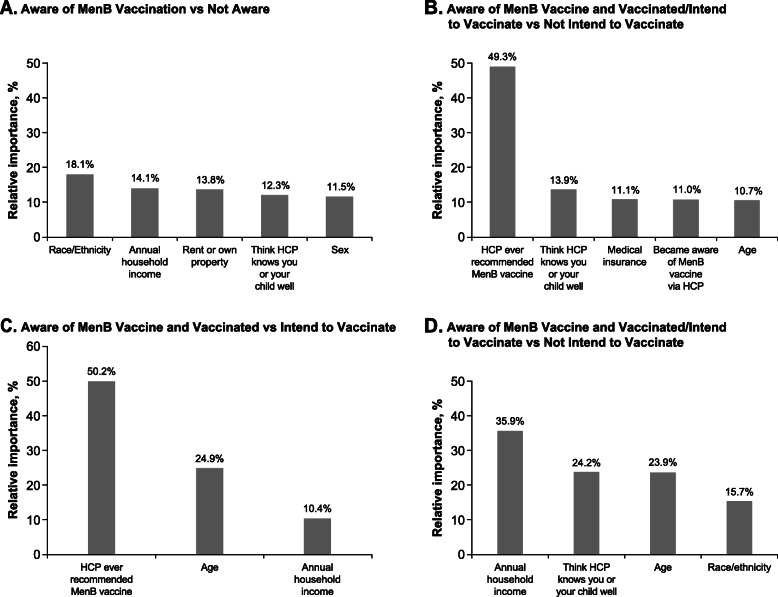
Relative importance of influential variables generated from CART for predicting MenB vaccine awareness and utilization. CART = Classification and Regression Tree; HCP = healthcare provider; MenB = meningococcal serogroup B

Multivariate analyses revealed that several demographic and social factors were significantly associated with MenB vaccine awareness, including sex (male vs female; odds ratio [OR] 0.43; 95% CI 0.26, 0.70), race (White, non-Hispanic vs Black and Others, non-Hispanic; OR 2.20; 95% CI 1.09, 4.46), and whether parents felt the HCP knew their child well (no vs yes; OR 0.53; 95% CI 0.30, 0.96) (Fig. 2a). Results from the CART analysis indicated that the most influential variable associated with awareness of MenB vaccines was race/ethnicity, with other associated variables being annual household income, property ownership, the feeling that their HCP knew their child well, and sex (Fig. 3a).

The majority of parents/guardians who were aware of MenB vaccines (69%) learned about the vaccines through an HCP; 16% were made aware by the media and the remaining 12% learned through another source, such as a family member, friend, coworker, or school (note that these choices were not mutually exclusive; data not shown).

#### Vaccinated/intention to vaccinate versus no intention to vaccinate

Results from univariate analyses indicated that a modestly higher percentage of parents and guardians who were aware of the MenB vaccine and vaccinated/intended to vaccinate their adolescents were Black/non-Hispanic, covered by employer-based medical insurance, aware of MenACWY vaccines or MenB outbreaks, or had higher educational attainment (some college or above), owned their own home, or felt their HCPs knew their child well, compared with parents who did not intend to vaccinate their adolescents (Table 1).

Multivariate analysis further supported that vaccinated/intention to vaccinate was significantly less likely if parents did not feel the HCP knew their child well (OR 0.43; 95% CI 0.20, 0.93), but significantly more likely if the provider had recommended the MenB vaccine (OR 4.81; 95% CI 2.46, 9.35) (Fig. 2b). Based on the CART analysis, a recommendation from a HCP was the most influential variable associated with vaccination/intention to vaccinate (compared with no intention to vaccinate; Fig. 3b). When respondents were asked how they became aware of the MenB vaccine, 82% of those who had vaccinated or intended to vaccinate their child were made aware of the MenB vaccine through their HCPs, compared with 48% of those with no intention to vaccinate (*P* < 0.0001; Table 2). Learning about the vaccine through the media was associated with the opposite effect (13% vs 26%; *P* = 0.001).

**Table 2:** Weighted results of MenB vaccine awareness and recommendation channels

	Aware and Vaccinated/ Intend to Vaccinate	Aware but Do Not Intend to Vaccinate	*P* Value	Aware and Vaccinated	Aware and Intend to Vaccinate	*P* Value
How did you first become aware of the MenB vaccine?						
Through HCP (e.g., physician or nurse)	74%	63%	**0.045**	80%	62%	**0.008**
Others (e.g., child, family member, friends, coworker, school)	14%	10%	0.429	13%	15%	0.629
Commercial (e.g., on TV, news in media)	13%	26%	**0.001**	7%	18%	**0.007**
HCP ever recommended MenB vaccine (yes)	82%	48%	**< 0.0001**	91%	64%	**< 0.0001**
Type of HCP who recommended MenB vaccine^a^						
Physician	90%	84%	0.272	90%	89%	0.809
Nurse	15%	11%	0.540	14%	15%	0.947
Medical assistant	1%	7%	**0.037**	1%	2%	0.611
Pharmacist	3%	1%	0.463	2%	7%	0.077
Other	3%	0%	0.202	4%	1%	0.324

*HCP* healthcare provider, *MenB* meningococcal serogroup B

^a^This survey question was given to a subset of responders who received a vaccine recommendation from their HCP

*P* < 0.05 is indicated in bold font to denote statistical significance

#### Vaccinated versus intention to vaccinate

Compared with parents/guardians who intended to vaccinate but had not yet done so, univariate analysis revealed modestly higher percentages of those who had vaccinated their adolescent children were female, Black/non-Hispanic, aware of the MenACWY vaccine, had employer-based medical insurance, or owned their home (Table 1).

**Fig. 4:**
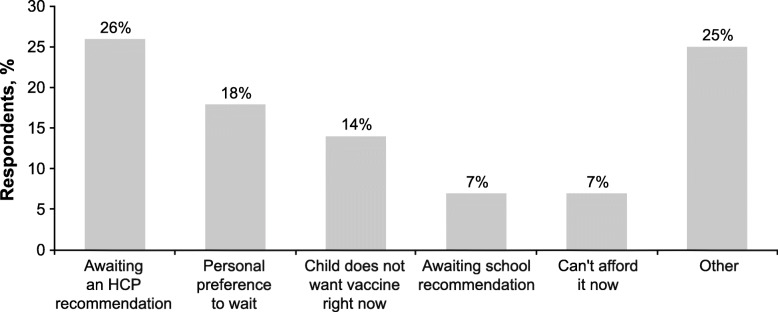
Common reasons provided for waiting to vaccinate. Data are for parents and guardians who intended to vaccinate but had not yet done so. HCP = healthcare provider

The results from the multivariate analysis indicated that those parents/guardians whose adolescent had already been vaccinated were significantly more likely to have some form of insurance, e.g., employer-based insurance (OR 3.34; 95% CI 1.09, 10.21) or other insurance (OR 3.66; 95% CI 1.06, 12.66); these parents were also more likely to have seen the same HCP consistently (OR 3.34; 95% CI 1.29, 8.62) and to have an HCP who had recommended a MenB vaccine (OR 5.66; 95% CI 2.49, 12.87) (Fig. 2c). Based on the CART analysis, a recommendation from a HCP was the most influential variable associated with vaccination (compared with intention to vaccinate; Fig. 3c). When parents/guardians who intended to vaccinate but had not yet done so were asked the reason for waiting, 26% responded that they had not yet received a recommendation from their HCP (Fig. 4). Personal preference to wait was indicated by 18% of respondents, followed by the child not wanting the vaccine yet (14%), waiting on a recommendation from the school (7%), and lack of affordability (7%).

#### Unaware but interested versus unaware and not interested

Of respondents previously unaware of the MenB vaccines, univariate analysis indicated that generally a higher percentage of parents or guardians interested in potentially vaccinating their children were Hispanic, aged ≥45 years, aware of MenACWY vaccines or MenB outbreaks, received Medicaid, had rented homes, or felt their HCPs knew their children well, compared with those who had no interest (Table 1).

Results from the multivariate analysis further supported that interest in MenB vaccination was significantly more common among Hispanic individuals (compared with non-Hispanic Black or non-Hispanic other; OR 5.05; 95% CI 1.13, 22.63) and those who were aware of the MenACWY vaccines (OR 3.02; 95% CI 1.03, 8.81) (Fig. 2d). Based on the CART analysis, among those who were unaware of MenB vaccines, the most influential factor associated with interest in learning about the vaccine was annual household income; other influential factors included the perception that their HCP knew their child well, age, and race/ethnicity (Fig. 3d).

## Discussion

MenB causes the majority of meningococcal disease cases among adolescents in the United States (greater than the number of serogroup A, C, W, and Y cases combined) [6], and meningococcal carriage rates are highest in this age group [8]. Ensuring equitable access to ACIP-recommended MenB vaccines is essential to help protect this vulnerable population, particularly in light of the ACIP’s non-routine, shared clinical decision-making (previously called “Category B” [19]) recommendation for MenB vaccines [21], the first recommendation to apply to an entire age group [29]. This study investigated parental and guardian awareness and utilization of MenB vaccines, revealing that the majority of parents/guardians were unaware of MenB vaccines and highlighting important racial and socioeconomic disparities in awareness and vaccination status; additionally, vaccination status or intention to vaccinate were strongly predicted by HCP-related factors.

### Lack of awareness among parents/guardians regarding MenB vaccines

The majority of parents and guardians (57%) in this study were unaware of MenB vaccination. These results are corroborated by a growing body of evidence on the impact of the shared clinical decision-making (Category B) recommendation on MenB vaccine awareness and utilization. In a 2017 survey of parents of high school students in Minnesota, 75.5% of parents were generally aware of the availability of meningococcal vaccines, but 31% were aware specifically of the MenACWY vaccine and only 18 to 20% were specifically aware of either licensed MenB vaccine [30]. Even fewer parents (7%) understood that the MenB vaccine helps protect against MenB disease, which is not covered by MenACWY vaccination. However, most parents were at least somewhat willing to vaccinate their adolescent children with MenB vaccines (90%), and intended to seek information from their providers about MenB vaccines (81%). The 2018 CDC NIS-Teen survey report indicated that nationwide only 17.2% of 17-year-olds in the United States had received 1 or more doses of the multidose MenB vaccine series [22], and only approximately half go on to complete the series [23]. By comparison, coverage rates for other adolescent vaccines administered in this age group were 86.6% (≥1 dose) and 50.8% (≥2 doses) for MenACWY, 51.1% (up-to-date doses) for the human papilloma virus (HPV) vaccine series, and 88.9% (≥1 dose) for the tetanus toxoid, reduced diphtheria toxoid, and acellular pertussis (Tdap) vaccine, among those aged 11 to 17 years [22].

### Socioeconomic and racial differences in MenB vaccine awareness and uptake

Our findings indicated that females and non-Hispanic, White individuals were significantly associated with increased MenB awareness among parents or guardians. Among parents or guardians unaware of MenB vaccines, there was significantly higher interest in the vaccine among parents of Hispanic ethnicity. Additionally, a marginally higher percentage of unaware respondents had insurance through Medicaid. Although insurance-related factors were not associated with awareness in our study, there is substantial precedent of insurance type-based disparities in healthcare and vaccine access among the US population, including among those patients insured through Medicaid [31–35].

These disparities in awareness are consistent with other studies examining factors associated with MenB vaccination among adolescents and young adults. In a cross-sectional study of 85,789 adolescents (aged 16–18 years) in the Philadelphia immunization registry between 2015 and 2017, only 16% had received ≥1 dose of a multidose MenB vaccine series, and 5% had completed the series [36, 37]. Multivariate analysis revealed that female sex, unknown or other reported race (compared with Black/African American race), and residing in a neighborhood with a median household income of greater than $100,000 were significantly associated with MenB vaccination, whereas Asian ethnicity was negatively associated with MenB vaccination. Additionally, in a retrospective cohort study using EHRs of 45,428 patients (aged 16–23 years) from 31 pediatric primary care practices in the Philadelphia region between 2015 and 2017, only 21% had received ≥1 dose of a multidose MenB vaccine [31]. Multivariate analyses revealed that MenB vaccine series completion was significantly associated with White race, having private insurance, and MenACWY vaccine receipt. As this study cohort comprised patients who had access to pediatric primary care, these results suggest that sociodemographic disparities likely persist regardless of access to healthcare.

### Healthcare provider impact on MenB vaccine awareness and uptake

Both the likelihood of having been vaccinated (compared with intention to vaccinate) and of being vaccinated/intending to vaccinate (compared with no intention to vaccinate) were most strongly predicted by factors directly related to the HCP. The importance of HCP recommendation was further supported by the CART results. More than a quarter of parents/guardians intending to vaccinate their adolescent were awaiting the recommendation from their HCP, and those who had first learned about MenB vaccines through their provider were significantly more likely to have vaccinated or have the intention of vaccinating their dependents.

In line with these results, previous research has indicated that parents expect providers to guide them on adolescent vaccines and cite the provider’s office as the most common information source for knowing when their adolescents’ vaccines are due [24]. The ACIP recommended that MenB vaccination be available to adolescents and young adults through provider-patient discussion and shared clinical decision-making (i.e., a Category B recommendation) [21], yet studies have shown that providers have a poor understanding of MenB vaccines and this ACIP recommendation [29, 36, 38–40]; Category B designation has paradoxically been shown to be the factor that hinders MenB vaccination [29, 38, 39]. In a nationally representative sample of US providers surveyed in 2016 by Kempe and colleagues, only 38% of family physicians and 56% of pediatricians were able to correctly define a Category B recommendation [29]. Many providers were also unsure about insurance coverage for a Category B vaccine, despite the fact that under the Affordable Care Act in the United States, all ACIP-recommended vaccines are covered by both private insurance plans and the CDC’s Vaccines for Children (VFC) program for all individuals 18 years and younger [41]. Ultimately, shared clinical decision-making requires discussion between provider and patients, which is impeded by providers’ lack of understanding of the ACIP recommendations.

Previous work also suggests a link between socioeconomic inequities and provider prescribing behavior. In a nationally representative survey, HCPs who prescribed MenB vaccines (compared with those who prescribed MenACWY only) were most likely to be seeing patients with private or commercial healthcare plans (i.e., not Medicaid) [40]. Additional findings from the aforementioned study from the Philadelphia region of 45,428 patients (aged 16–23 years) revealed lower MenB vaccine uptake among those seen in urban practice locations (vs suburban practice locations) [31]. This disparity may reflect different provider practices for MenB vaccine recommendations for different patient populations, and clinical-level purchasing decisions that impact rates of vaccine receipt.

Taken together, these data suggest a need to clarify the existing ACIP recommendation for MenB vaccines and to support providers and parents in the United States by developing consistent guidelines and concrete metrics that define a well-informed discussion for shared clinical decision-making. These efforts will help increase parental awareness of MenB vaccines and avoid reinforcing disparities in vaccine access and utilization that our study has uncovered, and ultimately ensure adolescents are comprehensively protected against meningococcal disease.

### Strengths and limitations

This survey had several strengths, including the use of the KnowledgePanel® as a data source, which is one of the most representative online panels in the United States, and has been successfully used in previous studies [25–27]. In addition, this study is one of the first national studies that corroborated findings from previously published regional studies evaluating MenB awareness [30]. Moreover, the use of address-based sampling and population-weighting methodology ensured data broadly represented the diverse US population. The panel also collected a wide range of demographic, social, and economic data that can be applied to survey analysis.

There were limitations of this study design relative to other studies involving online data collection, which included that the reported behaviors may not estimate actual behavior; for instance, the intention to vaccinate may not equate with vaccination. Vaccination statuses of approximately a third of participants were verified by EMR or HCP, as described in the Methods, to substantiate study results. Given that the survey was self-administered, some parents/guardians may have sought more information on the topic before completing the survey, leading to potential over reporting of MenB vaccine awareness. Finally, the study population may not be entirely representative of the US population as the sample size for some analyses was relatively small and KnowledgePanel® members may have been more inclined towards study participation, which could influence responses. However, as explained in the Methods, our study used population weighting to reduce biases from non-responders. Future analyses could further inform variables related to MenB vaccine uptake by including a stepwise regression to minimize the influence of HCP recommendation and to determine the remaining influential factors.

## Conclusion

A high percentage of parents and guardians of vaccine-eligible adolescents in the United States are unaware of MenB vaccines, with awareness influenced by racial and socioeconomic factors. Vaccination decisions by parents and guardians are highly reliant on the provider’s recommendation. Nevertheless, recently published studies demonstrate substantial gaps in provider understanding of MenB vaccine ACIP recommendations and raise concerns. Thus, to improve awareness among parents and guardians, provider understanding of the ACIP shared clinical decision-making (Category B) recommendation must be supported to aid consistent implementation of this recommendation for MenB vaccines. Overall, our data underscore the need for efforts to improve knowledge and awareness of MenB vaccines among parents and guardians.

## Data Availability

The study questionnaire and datasets generated and/or analyzed during the current study, which are not publicly available due to participant confidentiality, are available from the corresponding author on reasonable request.

## Abbreviations

ACIP: Advisory Committee on Immunization Practices
CART: Classification and regression tree
CDC: Centers for Disease Control and Prevention
EMR: Electronic medical record
HCP: Healthcare provider
HPV: Human papilloma virus
IMD: Invasive meningococcal disease
MenB: Meningococcal serogroup B
NIS-Teen: National Immunization Survey–Teen
OR: Odds ratio
Tdap: Tetanus toxoid, reduced diphtheria toxoid, and acellular pertussis
